# Non-Modified CpG Oligodeoxynucleotide Forming Guanine-Quadruplex Structure Complexes with ε-Poly-_L_-Lysine Induce Antibody Production as Vaccine Adjuvants

**DOI:** 10.3390/biom12121868

**Published:** 2022-12-13

**Authors:** Dandan Zhao, Anh Thi Tram Tu, Miwako Shobo, Nguyen Bui Thao Le, Chiaki Yoshikawa, Kazuhisa Sugai, Yoji Hakamata, Tomohiko Yamazaki

**Affiliations:** 1 Research Center for Functional Materials (RCFM), National Institute for Materials Science (NIMS), 1-2-1, Sengen, Tsukuba 305-0047, Japan; 2 Division of Life Science, Hokkaido University, Kita 10, Nishi 8, Kita-ku, Sapporo 060-0808, Japan; 3 Department of Magnetic and Biomedical Materials, Faculty of Materials Science and Technology, University of Science, 227 Nguyen Van Cu Street, Ward 4, District 5, Ho Chi Minh City 70000, Vietnam; 4 Ho Chi Minh City Campus, Vietnam National University, Linh Trung Ward, Thu Duc City, Ho Chi Minh City 70000, Vietnam; 5 School of Veterinary Nursing and Technology, Nippon Veterinary and Life Science University, 1-7-1 Kyonancho, Musashino-shi, Tokyo 180-8602, Japan

**Keywords:** guanine quadruplex, CpG oligodeoxynucleotides, ε-poly-_L_-lysine, immune stimulation, cytokine induction

## Abstract

Unmethylated cytosine-phosphate-guanosine oligodeoxynucleotides (CpG ODNs) induce inflammatory cytokines and type I interferons (IFNs) to activate the immune system. To apply CpG ODNs as vaccine adjuvants, the cellular uptake and stability of phosphodiester-based, non-modified ODNs require further improvement. Previously developed new CpG ODNs forming guanine-quadruplex (G4) structures showed higher nuclease resistance and cellular uptake than linear CpG ODNs; however, the complex formation of G4-CpG ODNs with antigen proteins is necessary for their application as vaccine adjuvants. In this study, we utilized a cationic polymer, ε-poly-_L_-lysine (ε-PLL), as a carrier for G4-CpG ODNs and antigen. The ε-PLL/G4-CpG ODN complex exhibited enhanced stability against nucleases. Cellular uptake of the ε-PLL/G4-CpG ODN complex positively correlated with the N/P ratio. In comparison to naked G4-CpG ODNs, the ε-PLL/G4-CpG ODN complex induced extremely high levels of interleukin (IL)-6, IL-12, and IFN-β. Relative immune cytokine production was successfully tuned by N/P ratio modification. Mice with the ε-PLL/G4-CpG ODN/ovalbumin (OVA) complex showed increased OVA-specific immunoglobulin (Ig)G, IgG1, and IgG2c levels, whereas total IgE levels did not increase and weight gain rates were not affected. Therefore, ε-PLL can serve as a safe and effective phosphodiester-based, non-modified CpG ODN delivery system, and the ε-PLL/G4-CpG ODN/antigen complex is a highly promising candidate for vaccine adjuvants and can be further used in clinical research.

## 1. Introduction

Oligodeoxynucleotides (ODNs), which contain unmethylated cytosine-phosphate-guanosine (CpG) motifs, have attracted considerable attention in recent years [1,2,3,4,5]. The unmethylated CpG ODN motifs are generally present in bacterial and viral DNA along with the toll-like receptor 9 (TLR9) and activate the innate and adaptive immune responses [6,7,8,9,10]. Toll-like receptor 9 has been considered a sensor for bacterial DNA [11]. After TLR9 combines with unmethylated CpG ODNs, the nuclear factor κB (NF-κB) and stress kinase pathways can be activated [12]. Activation of these two pathways results in the secretion of cytokines such as interleukin (IL)-6, IL-12, and tumor necrosis factor-α (TNF-α) [13,14]. Interleukin-6 strongly influences the production of antibodies and the development of effector T-cells [15], and IL-12 strongly affects the activation of antigen-specific T cells [16]. Therefore, CpG ODNs have excellent potential for use as vaccine adjuvants [17,18]. However, several barriers currently limit the clinical use of phosphodiester-based, non-modified CpG ODNs, such as their easy degradation by nucleases and inefficient cellular uptake [19,20].

A variety of approaches have been developed to increase the enzymatic stability of CpG ODNs. The phosphodiester backbone substituted by phosphorothioate can increase the enzymatic stability of CpG ODNs [21]. However, several side effects restrict the use of phosphorothioate-modified CpG ODNs, such as acute toxicity and nonspecific binding with proteins [22].

In our previous studies, we developed a structured CpG ODN named the guanine-quadruplex (G4)-based CpG ODN [23,24,25,26]. The presence of guanine-rich regions in G4-CpG ODNs allows linear CpG ODNs to easily form G4 structures in the presence of potassium and sodium ions, resulting in high nuclease resistance. Compared to linear CpG ODNs, G4-CpG ODNs induce high expression of cytokines in immune cells [24]. However, the immunostimulatory effect of G4-CpG ODN was limited in vivo. To activate the immune response, highly concentrated G4-CpG ODNs must be used [24]. To improve nuclease resistance and cellular uptake of G4-CpG ODNs, we used a positively charged liposome, 1,2-dioleoyl-3-trimethylammonium-propane lipid (DOTAP), as a carrier, resulting in greatly improved immunostimulatory effects of G4-CpG ODN on both mouse and human immune cells [26]. However, DOTAP is not suitable for practical use owing to its instability, heterogeneity, cytotoxicity, and high cost.

To address these limitations, we introduced a cationic polymer as a nanocarrier for delivering G4-CpG ODNs. Through electrostatic interactions, cationic polymers combine with CpG ODNs and neutralize the negative charge to increase the cellular uptake of CpG ODNs [27]. Among cationic polymers, polyethyleneimine (PEI) is one of the most studied gene delivery vectors [28,29,30]. Because PEI exhibits the proton sponge effect, it promotes DNA escape from the endosome [31]. Toll-like receptor 9 is located in the endosome; therefore, retaining CpG ODNs in the endosome is vital [32]. However, the proton sponge effect decreases the retention time of CpG ODNs in the endosome, further decreasing their activity. Therefore, PEI was unsuitable for the delivery of CpG ODNs. Moreover, PEI is nonbiodegradable and exhibits considerable cytotoxicity, which may cause cell apoptosis or organ damage. This restrictive factor inhibits the clinical application of PEI [33,34].

α-poly-_L_-lysine exhibits a negligible proton sponge effect, does not possess the proton buffering ability to enhance endosomal escape, and shows an almost equal percentage of localization efficiencies in the nucleus and lysosomes after forming polyplexes with plasmid DNA [35]. However, α-poly-_L_-lysine shows strong cytotoxicity [36]. Another type of polylysine, ε-poly-_L_-lysine (ε-PLL), has also attracted attention. ε-PLL, which is produced by microbial fermentation with *Streptomyces albulus*, is considered safe by the United States Food and Drug Administration (USFDA) and is widely used as a food preservative [37,38]. ε-PLL is a non-synthetic cationic polymer that is biodegradable, biocompatible, non-toxic, and highly organized [39]. Moreover, ε-PLL has the same monomer unit as α-poly-_L_-lysine, which demonstrates its strong potential as a DNA delivery system. Some experiments have shown that ε-PLL, with a molecular weight of approximately 4 kDa, can enhance IgM production in HB4C5 cells and interferon (IFN)-β production in human osteosarcoma MG-63 cells [40]. In addition, the price of ε-PLL is approximately 1000 times lower than the cost of other cationic lipid delivery systems, such as DOTAP, thereby increasing the attractiveness of ε-PLL as a G4-CpG ODN delivery system.

In this study, ε-PLL was used as a carrier for delivering G4-CpG ODNs. The negatively charged G4-CpG ODNs were condensed by the positively charged ε-PLL to form a nanoscale complex. Various ε-PLL/G4-CpG ODN complexes were formed at different N/P ratios to optimize their function. Dynamic light scattering, zeta potential measurements, and scanning electron microscopy (SEM) were used to characterize the size, charge, and morphology of the complexes, respectively. The serum stability of these complexes was estimated by polyacrylamide gel electrophoresis (PAGE). Flow cytometry and confocal fluorescence imaging were used to evaluate cellular uptake efficiency. For immunostimulatory evaluation, mouse macrophage-like RAW264 cells were used, and cytokine induction levels were quantified. Finally, we evaluated the vaccine adjuvant effect of ε-PLL/G4-CpG ODN/ovalbumin (OVA) complexes in mice.

## 2. Materials and Methods

### 2.1. Materials

A 25% (*w/w*) ε-poly-_L_-lysine (ε-PLL) (average molecular weight: 4000) aqueous solution was purchased from JNC Corp. (Tokyo, Japan), which was used without purification. Dulbecco’s phosphate-buffered saline (D-PBS), containing 2.68 mM KCl, 137 mM NaCl, 1.47 mM KH_2_PO_4_, and 8.10 mM Na_2_HPO_4_, was purchased from DS Pharma Biomedical (Osaka, Japan). The cell counting kit 8 (CCK-8) was purchased from Dojindo (Kumamoto, Japan). Unlabeled guanine-quadruples (G4)-CpG oligodeoxynucleotides (ODN) with a backbone comprising entirely of phosphodiester, named GD3 (5′-GGGTTGGGGTCGTTTTGTCGTTTTGTCGTTGGGTTGGG-3′, 38base), G4-GpC ODN, a CG sequence inverted to a GC sequence, named GD3_GpC (5′-GGGTTGGGGTGCTTTTGTGCTTTTGTGCTTGGGTTGGG-3′, 38base), linear-CpG ODN, with the same sequence as that of the CpG motif inserted into the 2nd loop of GD3, named G0075 (5′-GTCGTTTTGTCGTTTTGTCGTT-3′, 22base), ssODN38mer (5′-GTCGTTTTGTCGTTTTGTCGTTTTGTCGTTTTGTCGTT-3′; 38 base), and GD3 labeled with Cy5 at the 5′ end (G4-CpG ODN^5′Cy5^) were purchased from Eurofins Genomics (Tokyo, Japan). All CpG ODNs were of high-performance liquid chromatography purity grade and contained a backbone comprised entirely of a phosphodiester sequence.

### 2.2. G4 Structure Formation

The G4 structure was formed according to a previously reported method [24]. Briefly, to obtain 100 μM ODNs, 40 μL of ODN stock solution (250 μM) was mixed with 10 μL of 10× D-PBS and 50 μL of sterile Milli-Q water in a PCR tube. The PCR tube was vortexed, spun down and placed in a thermal cycler (PCR Thermal Cycler Dice Standard TP650, Takara Bio, Shiga, Japan). The solution was then heated to 95 °C for 5 min and subsequently cooled slowly to 30 °C for 30 min, followed by cooling of the solutions to 4 °C. The formed G4-CpG ODNs solution was stored at 4 °C until use and then diluted to the indicated concentration with 1× D-PBS before use.

### 2.3. Preparation of the ε-PLL/G4-CpG ODN Complexes

The N/P ratio is an important characteristic of cationic polymer/nucleic acid complexes. The N/P ratio describes the molar ratio of amine nitrogen groups (N) in ε-PLL to nucleic acid phosphate groups (P) in the ODNs. N is calculated by the amount of ε-PLL/average molecular weights (MWs) of repeating units of ε-PLL (MW of lysin: 128 g/mol), whereas P is calculated by the amount of OPN/average MWs of repeating units of ODNs (MW of nucleotide: 330 g/mol).

The desired amounts of ε-PLL (0.12, 0.24, 0.36, 0.48, 0.6, 1.2, and 2.4 mg for an N/P of 1, 2, 3, 4, 5, 10, and 20, respectively) were diluted to a final volume of 1 mL in 1× D-PBS. The ε-PLL solutions were mixed with an equal volume of 25 μM G4-CpG ODN solution and immediately mixed thoroughly by pipetting. After mixing, the solution was incubated at 25 °C for 10 min. The concentration of CpG ODN in the complex solution was fixed at 12.5 μM. The final concentrations of the G4-CpG ODNs and ε-PLL in each solution are summarized in Table 1.

### 2.4. Particle Size, Surface Charge, and Morphological Analysis of the ε-PLL/G4-CpG ODN Complexes

The hydrodynamic sizes of the complexes were measured using dynamic light scattering (DLS-8000; Otsuka Electronics, Osaka, Japan) with a He-Ne laser (633 nm). The average particle size was calculated by number-weighted particle size distributions using the Marquardt algorithm. The zeta potentials of the complexes were measured using a zeta potential analyzer (ELSZ-1000; Otsuka Electronics). Ultraviolet-visible (UV-Vis) absorption spectra were recorded on a NanoDrop 2000 spectrophotometer (Thermo Fisher Scientific, Waltham, MA, USA). A 1.5 μL portion of the sample solution was dropped onto the sample stage, and the absorbance was read from 200 nm to 500 nm. Scanning electron microscopy images were obtained using a TM3000 Tabletop Microscope (Hitachi, Tokyo, Japan) and a high-resolution scanning electron microscope, SU-8000 (Hitachi, Tokyo, Japan). To avoid agglomerates, the complex was well dispersed by ultrasonication, and the diluted solution was then placed on a silicon wafer and air-dried slowly overnight. Milli-Q water was used to wash the samples for desalting. Before SEM visualization, the samples were coated with osmium using an osmium plasma coater (Filgen, Nagoya, Japan).

### 2.5. Cell Culture

The mouse macrophage-like cell line RCB053, named RAW264 (RIKEN Bio Resource Center, Tsukuba, Japan), was maintained in a minimum essential medium (MEM, Thermo Fisher Scientific) containing 2 mM l-glutamine, 10% (*v/v*) fetal bovine serum (FBS) (Sigma-Aldrich, St Louis, MO, USA), and 1% (*v/v*) MEM non-essential amino acid solution (100×; Fujifilm Wako Pure Chemical, Osaka, Japan). The cells were maintained in a humidified incubator with 5% CO_2_ at 37 °C. For the assay, penicillin and streptomycin mixtures (Thermo Fisher Scientific) were added to the cell culture medium to final concentrations of 100 units/mL and 100 µg/mL, respectively.

### 2.6. Cytotoxicity Assay

RAW264 cells were seeded in 96-well plates at a density of 5000 cells/well (in 180 μL of medium). After incubation for 24 h in a humidified incubator with 5% CO_2_ at 37 °C, 20 μL of ε-PLL solution dissolved in 1× D-PBS was added to the culture medium. After another 24 h of incubation, 10 μL of CCK-8 reagent was added to the medium, which was further incubated for 4 h with 5% CO_2_ at 37 °C. Absorbance at 450 nm was measured using a microplate reader (MTP-880 Lab; Corona Electric, Ibaraki, Japan). The cell viability was calculated using the formula below. The medium without cells was used as a blank. Cells in which only 1× D-PBS buffer was added were used as controls.

$$\%viable\;cells=\frac{\left(absorbance\;of\;sample-absorbance\;of\;blank\right)\;}{\left(absorbance\;of\;control-absorbance\;of\;blank\right)}\times 100\%$$

### 2.7. Stability of G4-CpG ODN Complexed with ε-PLL in Serum

The stability of G4-CpG ODN in the complexes was confirmed by PAGE. Naked 12.5 μM G4-CpG ODN or ε-PLL/G4-CpG ODN complexes (both at 3 μL) at different N/P ratios were mixed with 27 μL of 1× D-PBS containing 55.6% (*v/v*) FBS to obtain a final concentration of 50% (*v/v*) in the reaction mixture. The mixture was incubated at 37 °C for 1, 2, 4, and 24 h. After the incubation, 3 μL ethylenediaminetetraacetic acid (EDTA 0.25 M) was added, and the mixture solution was heated to 80 °C for 2 min to quench the reaction. Subsequently, the samples were stored at 4 °C until the PAGE analysis. Serum-treated samples (6.5 μL) were applied to 10–20% linear gradient polyacrylamide gels (e-PAGEL gels; ATTO, Tokyo, Japan). Polyacrylamide gel electrophoresis was performed in Tris-glycine buffer (25 mM Tris, 192 mM glycine, pH 8.3) at a constant current of 21 mA for 65 min. An ultralow-range DNA ladder (Thermo Fisher Scientific) was used as a marker. The gel was stained with SYBR^®^ gold nucleic acid gel stain (Thermo Fisher Scientific) for 40 min. Quantification of non-degraded G4-CpG ODN was performed by determining the fluorescence intensity of the band in the gel using Image Studio Lite version 5.2 (Li-COR Biotechnology, Lincoln, NE, USA).

### 2.8. Cellular Uptake

Flow cytometry was performed to quantitatively evaluate the cell uptake efficiency. RAW264 cells were seeded in a 48-well plate at a density of 2 × 10^5^ cells/well (in 200 μL of medium) and then incubated for 18 h at 37 °C. Seventy-two microliter samples of 12.5 μM naked G4-CpG ODN^5′Cy5^ or the ε-PLL/G4-CpG ODN^5′Cy5^ complex were mixed with 128 μL Opti-MEM (Thermo Fisher Scientific). The culture medium was replaced with 200 μL of the fluorescently labeled CpG ODN solution prepared above. After 2 h of incubation at 37 °C or 4 °C, the cells were washed twice with 500 μL of phosphate-buffered saline (PBS; Fujifilm Wako Pure Chemical, Osaka, Japan). The cells were harvested by adding 500 μL of trypsin (500 mg/mL)—EDTA (200 mg/L) solution (Thermo Fisher Scientific) and incubated at 37 °C for 30 min. The cells were then collected via centrifugation at 500× *g* for 10 min and washed once with 500 μL of PBS before being resuspended in 500 μL PBS. The mean fluorescence intensity (MFI) was measured by a flow cytometer (SP6800, Sony, Tokyo, Japan).

Confocal microscopy was used to visualize the cellular uptake. RAW264 cells (1.2 × 10^5^) were seeded in a cell culture dish with a glass bottom (CELLview; Greiner Bio-One, Kremsmünster, Austria) coated with collagen before use. To coat the dish, 200 μL of collagen coating solution (Cell Applications Inc., San Diego, CA, USA) was added to the dish and incubated at 25 °C for 1 h. After incubation for 18 h, naked G4-CpG ODN^5′Cy5^ and the ε-PLL/G4-CpG ODN^5′Cy5^ complex were added separately to the dish at a final ODN concentration of 4.5 μM. After incubation at 37 °C for 2 h, cells were washed with PBS and fixed with 100% methanol, followed by the addition of 4% (*v/v*) paraformaldehyde dissolved in PBS. Two drops of slow-fade diamond antifade mountant with DAPI (Thermo Fisher Scientific) were added to stain the nuclei. The fluorescence in the cells was visualized using a confocal laser scanning microscope (TCS SP5 II; Leica Microsystems, Wetzlar, Germany). The software LAS AF version 2.6.3 (Leica Microsystems) was used to process the images.

### 2.9. Immunostimulatory Stimulation Assay in RAW264 Cells

To determine the relative transcript levels of IL-6, IL-12, and IFN-β, RAW264 cells were seeded into a 96-well plate at a density of 1 × 10^5^ cells/well (5.5 × 10^5^ cells/mL, 180 μL). After incubation for 18 h, 20 μL of naked G4-CpG ODN or ε-PLL/G4-CpG ODN complexes with different N/P ratios were added to the 96-well plate. The final concentration of G4-CpG ODN in the medium was 1.25 μM. The cells were then incubated for 24 h, and the reagent ISOGEN (Nippon Gene, Tokyo, Japan) was used to extract RNA. The extracted mRNA was treated with DNase I (RNase-free) (Takara Bio) and subsequently purified using magnetic beads (RNAClean XP; Beckman Coulter, Brea, CA, USA). The mRNA (0.5 μg) was converted to cDNA using reverse transcriptase (Takara Bio). Quantitative real-time RT-PCR was performed using 50 ng of cDNA and the primer (FASMAC, Kanagawa, Japan) incorporating LightCycler480 SYBR Green I Master (Roche, Basel, Switzerland) in a Light Cycler 2.0 system (Roche). The mRNA levels were normalized to those of glyceraldehyde 3-phosphate dehydrogenase (GAPDH).

The primers used were as follows: GAPDH: forward, 5′-GTG GAC CTC ATG GCC TAC AT -3′ and reverse, 5′-TGT GAG GGA GAT GCT CAG TG -3′; IL-6: forward, 5′-TCC TTC CTA CCC CAA TTT CC -3′ and reverse, 5′-CGC ACT AGG TTT GCC GAG TA -3′; IL-12: forward, 5′-GAA AGG CTG GGT ATC GG -3′ and reverse, 5′-GGC TGT CCT CAA ACT CAC -3′; and IFN-β: forward, 5′- GGT CCG AGC AGA GAT CTT CA -3′ and reverse, 5′- TCA CTA CCA GTC CCA GAG TCC -3′.

### 2.10. Immunization

Five-week-old male C57BL/6 mice were obtained from the Charles River Laboratory Japan (Yokohama, Japan). Animal studies were approved by the Animal Care and Use Committee at the National Institute for Materials Science according to the Guidelines for Proper Conduct of Animal Experiments established by the Science Council of Japan. After 1 week of acclimation in a pathogen-free environment, 200 μL of vaccine solution containing 200 μg of ovalbumin (OVA; Sigma-Aldrich) alone or a mixture with 50 μg of G4-CpG ODN complexed with 2000 μg of ε-PLL was subcutaneously injected into the back twice at 1-week intervals. The amounts of G4-CpG ODN and ε-PLL were adjusted to an N/P ratio of 10. Blood samples were drawn from a vascular bundle located just above the rear of the jawbone using an animal lancet (MEDIpoint, Mineola, NY, USA), placed in a blood collection tube containing a clot activator and separation gel (Terumo, Tokyo, Japan), and centrifuged at 3000× *g* for 10 min at 4 °C. The resulting serum was stored at −20 °C until analysis.

### 2.11. Detection of OVA-Specific Antibodies via Enzyme-Linked Immunosorbent Assay (ELISA)

Ovalbumin-specific serum antibodies, OVA-total IgG, OVA-IgG1, and OVA-IgG2c, were measured using ELISA. The plates were coated with OVA at a concentration of 10 μg/mL in 1× PBS overnight at 4 °C. The plates were washed five times in 1× PBS containing 0.05% (*v*/*v*) Tween 20 (PBST) using a microplate washer (Model 1575, BioRad, Hercules, CA, USA), and blocked with the blocking reagent N102 (NOF, Tokyo, Japan) diluted 1:5 in pure water for 1 h at 25 °C. The plates were then washed five times with PBST. Mouse anti-OVA monoclonal IgG1 and IgG2c antibodies (Chondrex, Woodinville, WA, USA) or serum diluted in ChonBlock ELISA buffer (Chondrex) were added to the wells, and the plates were incubated for 2 h at 25 °C. After the plates were washed five times with PBST, horseradish peroxidase samples conjugated with anti-mouse IgG, IgG1, or IgG2c polyclonal antibodies (Bethyl, Montgomery, TX, USA) at a concentration of 10 ng/mL in Can Get Signal Immunoreaction Enhancer Solution 2 (Toyobo, Osaka, Japan) were added and incubated at 37 °C for 1 h. The plates were washed 10 times in PBST, 3,3′,5,5′-tetramethylbenzidine (TMB) ready-to-use solution (Fujifilm Wako Pure Chemical) was added, and the plates were incubated for 15 min. The reaction was stopped with 1 M sulfuric acid, and the absorbance was read at 450 nm. Total IgE was measured using an IgE mouse uncoated ELISA kit (Thermo Fisher Scientific), according to the manufacturer’s instructions.

### 2.12. Statistical Analysis

Statistical analysis was performed using GraphPad Prism version 8.2.0 (GraphPad Software, La Jolla, CA, USA). A one-way analysis of variance (ANOVA) with Tukey’s multiple comparison test was used.

## 3. Results

### 3.1. Preparation and Characterization of the ε-PLL/CpG ODN Complexes

**Figure 1:**
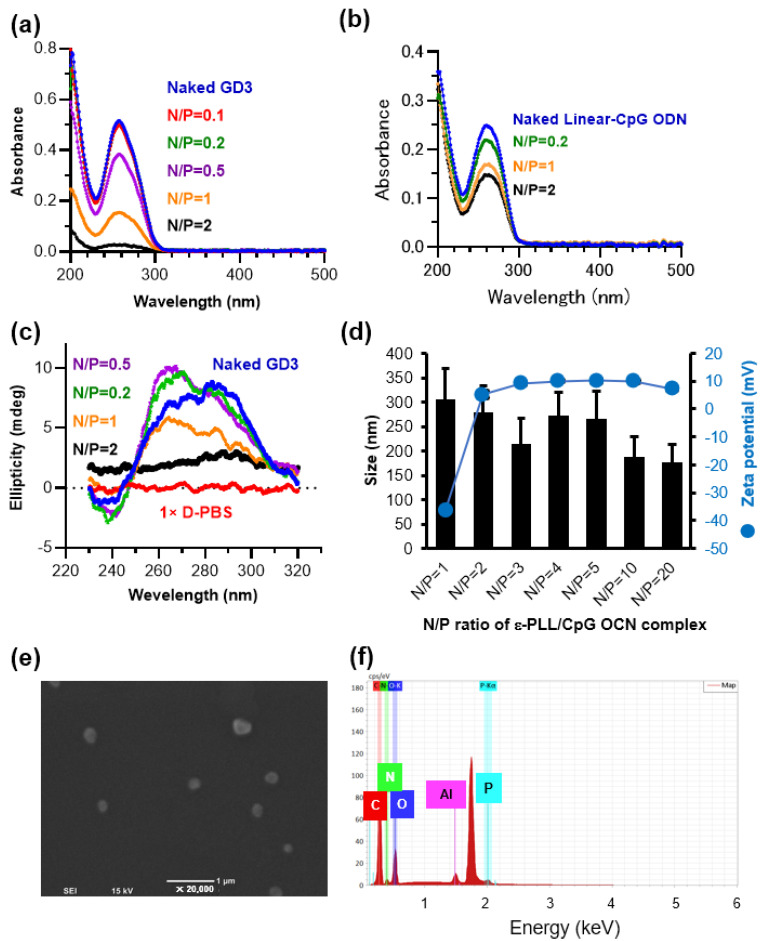
Characterization of the ε-PLL/G4-CpG ODN complexes. (**a**) Ultraviolet-visible (UV-Vis) absorption spectra of G4-CpG ODN (blue, 12.5 μM) and ε-PLL/CpG ODN at N/P ratios of 0.1 (red), 0.2 (green), 0.5 (purple), 1 (orange), and 2 (black). (**b**) UV-vis absorption spectra of linear-CpG ODN (blue, 12.5 μM) and ε-PLL/linear-CpG ODN at N/P ratios of 0.2 (green), 1 (orange), and 2 (black). (**c**) Circular dichroism spectra of naked G4-CpG ODN, GD3 (red), and ε-PLL/G4-CpG ODN at N/P ratios of 0.2 (green), 0.5 (purple), 1 (orange), 2 (black), and 1× D-PBS buffer (blue). (**d**) Zeta potential and hydrodynamic size of ε-PLL/G4-CpG ODN at different N/P ratios. Data are presented as mean ± SD (*n* = 3). (**e**) Scanning electron microscopy image of ε-PLL/G4-CpG ODN at an N/P ratio of 10. (**f**) Energy dispersive X-ray spectroscopic analysis of the ε-PLL/G4-CpG ODN complexes at an N/P ratio of 10.

The ε-PLL/CpG ODN complexes were prepared by mixing the polymer solution with an equal volume of the CpG ODN solution. In the present study, we used a guanine quadruplex structure-based CpG ODN (G4-CpG ODN) named GD3, which contains three CpG motifs in the central loop region of the G4 structure and showed the highest immunostimulatory effect among the G4-CpG ODNs we constructed [24]. Although G4-CpG ODNs are unmodified ODNs, they acquire resistance to nucleases because of their compact structure. As a result, compared to linear-CpG ODNs, G4-CpG ODNs showed excellent immunostimulatory activity in immune cells and mice [23,24,25,26]. The electrostatic interaction between the positively charged amino group on ε-PLL and the negatively charged phosphate group on G4-CpG ODN caused ODN condensation into the complexes. The UV-vis absorption spectra of naked G4-CpG ODN and ε-PLL/G4-CpG ODN complexes at N/P ratios ranging from 0.1 to 2 are shown in Figure 1a. The naked G4-CpG ODN showed the characteristic absorbance of the oligonucleotide peak at 260 nm, which was not observed in ε-PLL (Figure S1). The absorbance of ε-PLL/G4-CpG ODN complexes at 260 nm decreased as the N/P ratio increased. At N/P ratios of 0.1 and 0.2, the absorbance at 260 nm was not substantially different between the naked G4-CpG ODNs and ε-PLL/G4-CpG ODN complexes. After the N/P ratio reached 0.5, a decrease in the absorbance at 260 nm was observed. A further decrease was observed at an N/P ratio of 1, and at an N/P ratio of 2, the absorbance at 260 nm was less than 10% that of bare G4-CpG ODNs. The addition of the ε-PLL solution leads to a compact G4-CpG ODN chain, and solid particles may form and precipitate from the solution. Therefore, the absorbance at 260 nm decreases [33]. Most G4-CpG ODNs form a complex at an N/P ratio of 2. In addition, we prepared complexes using linear-CpG ODN (Figure 1b). At 260 nm, the absorbance of ε-PLL/linear-CpG ODN complexes decreased as the N/P ratio increased, similar to that observed in G4-CpG ODNs. Compared to ε-PLL/G4-CpG ODNs, the absorbance of ε-PLL/linear-CpG ODNs was high at the same N/P ratio. Even at an N/P ratio of 2, free linear-CpG ODNs remained in a 40% solution. These results indicate that the G4 structure promotes complex formation with ε-PLL and ODNs more than the linear structure.

The effect of ε-PLL on the G4 topology of G4-CpG ODNs was also investigated. Figure 1c shows the circular dichroism (CD) spectra of naked G4-CpG ODN and ε-PLL/G4-CpG ODN complexes at N/P ratios ranging from 0.2 to 2. The CD spectrum of the complex at an N/P ratio of 2 was similar to that of the 1× D-PBS buffer, providing no information on the structure of G4-CpG ODNs in the complex. Because the G4-CpG ODNs in the complex did not show absorbance (Figure 1a), it was not possible to measure the CD spectrum of G4-CpG ODNs in the complex. To estimate the structure of the G4-CpG ODNs in the complex, we measured the CD spectra of ε-PLL/G4-CpG ODN complexes at N/P ratios of 0.2, 0.5, and 1. The results showed that G4-CpG ODNs mixed with ε-PLL retained the hybrid-type topology, with positive peaks at 265 nm and 295 nm, similar to naked G4-CpG ODN. However, the height of the peak at 265 nm, which was derived from the parallel-type topology, increased, indicating that the topology of some G4-CpG ODNs was parallel. Therefore, we inferred that ε-PLL contributes to the change in the topology of G4-CpG ODN to parallel and that G4-CpG ODN forms both parallel and hybrid topologies in the complex.

Next, we performed PAGE analysis using Tris-glycine buffer containing 4 mM potassium in the running buffer to estimate the stability of ε-PLL/G4-CpG ODN complexes and the molecularity of G4-CpG ODNs in the complexes. As shown in Figure S2, bare G4-CpG ODN separated as three distinct bands on the gel, indicating that the complex adopts three distinct structures of different hydrodynamic radii with different folding topologies. G4-CpG ODN-eluted ε-PLL/G4-CpG ODN complexes showed the same migration pattern as bare G4-CpG ODN. The fluorescence intensity of G4-CpG ODN eluted from the complex by electrophoresis decreased as the N/P ratio of the complex increased. The fluorescence intensities of the G4-CPG ODN bands in the complexes at N/P ratios of 2, 5, and 10 were very weak. The G4-CpG ODNs were not eluted from the complexes, indicating that the ε-PLL/G4-CpG ODN complex was stable in the presence of potassium ions.

The hydrodynamic size and zeta potential strongly affect cellular uptake and cytotoxicity; therefore, the hydrodynamic size and zeta potential of ε-PLL/G4-CpG ODN complexes were determined at various N/P ratios. As shown in Figure 1d, the average size of the complexes decreased with increasing N/P ratios. At an N/P ratio of 1, the average ε-PLL/G4-CpG ODN complex size was approximately 300 nm, which decreased to 160 nm at an N/P ratio of 20. The polydispersity index of the ε-PLL/G4-CpG ODN complex at N/P ratios of 1 to 20 ranged between 0.03 to 0.18 (Table S1). We also calculated the intensity-, volume-, and number-weighted particle sizes using the Marquardt algorithm (Figure S3). These results indicated that the complexes exhibited a slightly broad dispersion. The ε-PLL/G4-CpG ODN complexes at an N/P ratio of 1 exhibited a highly negative charge (−36 mV), and upon increasing the N/P ratio to 2, the complexes showed a positive charge of approximately +5 mV. However, there was no significant increase in the zeta potential values with further increases in the N/P ratio. At an N/P ratio of 1, the highly negative charge was caused by the presence of a larger amount of free naked G4-CpG ODN. Moreover, a highly negative charge results in strong electrostatic repulsion, which leads to large average complex sizes at low N/P ratios. The electrostatic interaction between the positively charged amino group on ε-PLL and the negatively charged phosphate group on G4-CpG ODN was positively correlated with the N/P ratio, which led to a decrease in the average size of the complexes [33,41]. Therefore, when the N/P ratio reached 20, the average complex size was smaller than that at an N/P ratio of 1. These results indicate that stable particles can be formed by mixing ε-PLL and G4-CpG ODN at an N/P ratio of 2 or higher.

Scanning electron microscopy was used to study the size and morphology of the complexes. Figure 1e shows the SEM image of the complexes at an N/P ratio of 10. The average nanoparticle size in the SEM image was 262 nm (SD = 42 nm), which was consistent with the results of the dynamic light scattering analysis. Energy dispersive X-ray spectroscopic analysis detected C, O, N, and P in the complexes, as shown in Figure 1f. The peak of phosphorus atoms derived from nucleic acids was observed, suggesting that G4-CpG ODNs are contained in the complex. The morphologies of the complexes were analyzed using high-resolution SEM. The results showed that most of the particles were 100–300 nm in size and heterogeneous in shape (Figure S4). In addition, aggregates of multiple complexes were observed.

### 3.2. Cytotoxicity of ε-PLL

**Figure 2:**
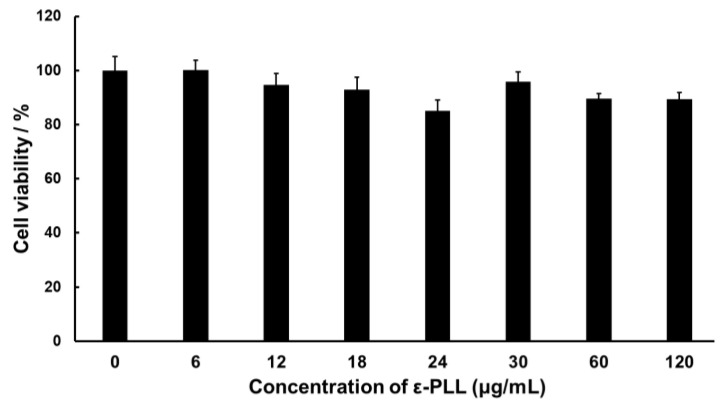
Viability of RAW264 cells treated with various concentrations of ε-PLL. Data represent the mean ± SD (*n* = 5).

The cytotoxicity of ε-PLL in RAW264 cells was evaluated. Figure 2 shows the cell viability results for various concentrations of ε-PLL. The concentration of the ε-PLL solution shown in Figure 2 is related to the complex solution, with N/P ratios ranging from 1 to 10. We found that ε-PLL did not induce substantial cytotoxicity. This result indicates that the ε-PLL solution exhibited high biocompatibility and could be safely used as a CpG ODN carrier.

### 3.3. Nuclease Resistance of G4-CpG ODNs in Complex

**Figure 3:**
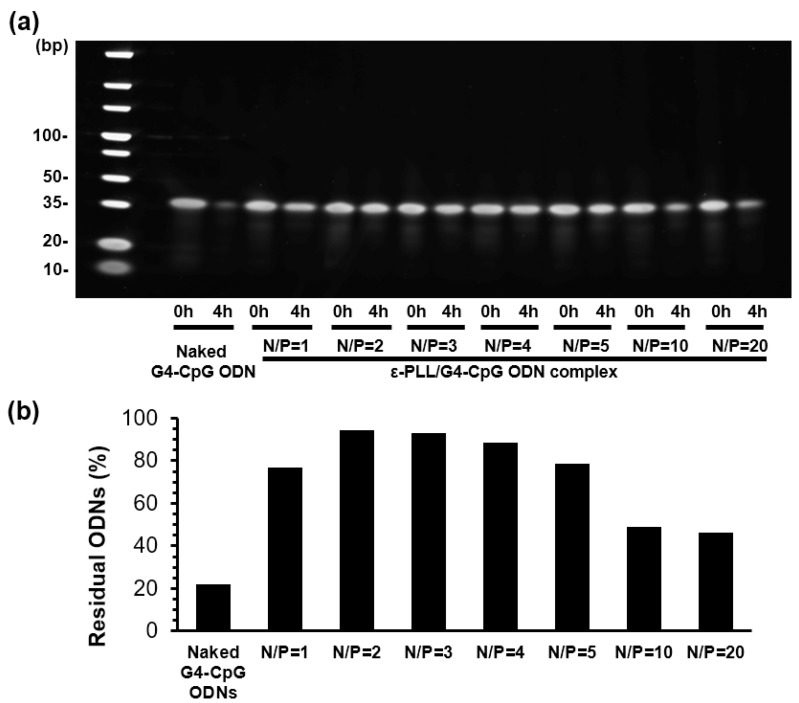
Nuclease resistance of naked G4-CpG ODN and G4-CpG ODN in ε-PLL/G4-CpG ODN complexes at various N/P ratio in 50% fetal bovine serum after incubation for 0 and 4 h. (**a**) Image of polyacrylamide gel electrophoresis. (**b**) Quantitative results of G4-CpG ODNs remaining after incubating in 50% serum for 4 h.

Nuclease resistance is one of the greatest barriers to the successful use of CpG ODN in vitro and in vivo. In this study, the nuclease resistance of ε-PLL/G4-CpG ODN complexes was compared with that of naked G4-CpG ODN with 50% (*v/v*) of FBS at 37 °C. The potassium and sodium ion concentrations in 1× D-PBS buffer were the same as those in the serum; therefore, the final concentrations of potassium and sodium ions in the assay solution were 4 mM and 150 mM, respectively. We previously reported that compared to linear-CpG ODNs, G4-CpG ODNs have enhanced stability in serum because their compact structure prevents degradation by nucleases. Figure S5 shows the PAGE images of naked G4-CpG ODN and ε-PLL/G4-CpG ODN after incubation with serum for 1, 2, 4, and 24 h. The fluorescence intensity of the bands indicates the amount of residual G4-CpG ODN. We observed that 80% of the naked G4-CpG ODNs were degraded within 4 h, whereas bands of G4-CpG ODNs in complexes remained almost completely intact after incubation for 4 h at all dose ratios. To clearly show the effect of the N/P ratio on nuclease resistance, complexes with N/P ratios of 1 to 20 were incubated with serum for 4 h in a single gel to quantify the amount of residual G4-CpG ODN (Figure 3a). We used a potassium-ion-free running buffer for PAGE. In the absence of potassium ions, the G4 structure of G4-CpG ODN was unstable and changed rapidly to a linear structure. As shown in Figure S1, the interaction between linear-CpG ODN and ε-PLL was weak; therefore, ODNs were eluted from the complex by electrophoresis. Figure 3b shows that the complex with an N/P ratio of 2 showed the highest nuclease stability, with the remaining 95% of the G4-CpG ODN remaining undegraded. No significant decreases were observed at N/P ratios of 1, 2, 3, 4, and 5, and nearly 80% of G4-CpG ODN remained in all samples. However, as the N/P ratio increased to 10 or 20, the percentage of residual G4-CpG ODN decreased to 50%. This may be because of the additional free cationic polymer. Gary et al. confirmed that an abundance of free cationic polymer leads to cationic polymer binding, with negatively charged DNA being reversible [42]. Therefore, the complexes formed at high N/P ratios lack stability. This result suggests that the nuclease resistance of G4-CpG ODN increased after condensation by ε-PLL.

### 3.4. Cellular Uptake by the ε-PLL/G4-CpG ODN Complexes

**Figure 4:**
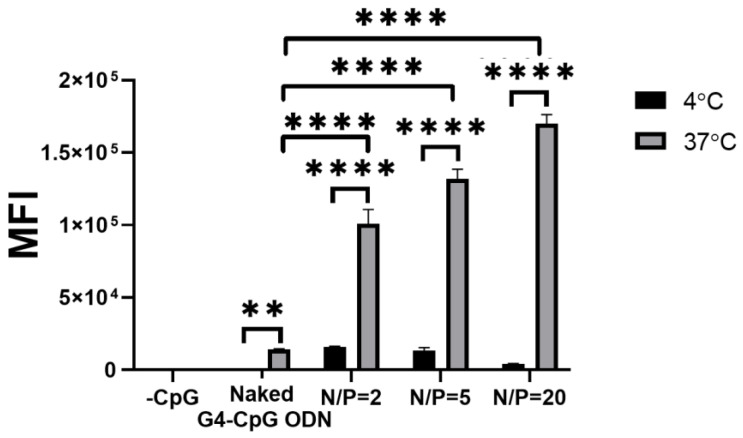
Flow cytometry analysis of cellular uptake by Cy5-labeled G4-CpG ODN and ε-PLL/G4-CpG ODN^5′Cy5^ complexes in RAW264 cells. The final G4-CpG ODN^5′Cy5^ concentration was 4.5 μM. Data represent mean ± SD (*n* = 3). In the graph, **** *p* < 0.0001, ** *p* < 0.01 (one-way ANOVA and Tukey’s multiple comparisons test).

To quantitatively estimate cellular uptake efficiency, the uptake of Cy5-labeled G4-CpG ODNs in RAW264 cells was quantified using flow cytometry. The MFI was calculated from histograms displaying the fluorescence intensity of cells treated with G4-CpG ODN^5′Cy5^ and ε-PLL/G4-CpG ODN^5′Cy5^ complexes (Figure S6). As shown in Figure 4, the MFI of RAW264 cells incubated with the ε-PLL/G4-CpG ODN complex was markedly higher than that of cells incubated with naked CpG ODNs at 37 °C. The MFI of RAW264 cells positively correlated with the N/P ratio of the ε-PLL/G4-CpG ODN complex. The formation of the ε-PLL/G4-CpG ODN complex increased the cellular uptake of G4-CpG ODN.

**Figure 5:**
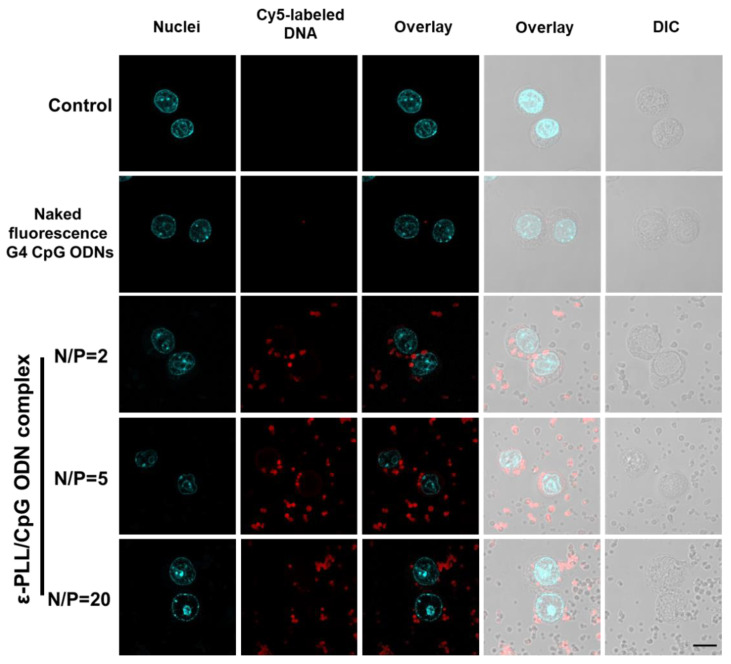
Confocal microscopy image of cellular uptake by the ε-PLL/G4-CpG ODN^5′Cy5^ complexes in RAW264 cells. The nuclei of cells were stained with DAPI (cyan). G4-CpG ODN is labeled with Cy5 (red). Scale bar: 10 μm. Non-treated RAW264 cells were used as the negative control. DIC, differential interface contrast observation.

Confocal microscopy was used to visualize the intracellular localization of the ε-PLL/G4-CpG ODN complex. The optical sections in confocal scanning microscopy were approximately 0.5 μm in thickness with a 64× objective lens. Therefore, only the ε-PLL/G4-CpG ODN complexes existing within 0.5 μm thickness from the focal point exhibited red fluorescence. In contrast, differential interface contrast observations reveal all particles on the optical path. Therefore, although some particles did not show fluorescence, all particles incorporated Cy5-labeled G4-CpG ODN. As shown in Figure 5, bright intracellular red fluorescence was observed in RAW264 cells treated with the ε-PLL/G4-CpG ODN^5′Cy5^ complex, whereas the weak fluorescence in the cells incubated with naked G4-CpG ODN^5′Cy5^ pointed to a low cellular uptake. This result indicated that the complexes engaged in high cellular uptake efficiency and successfully delivered G4-CpG ODN into the cells. The charge difference between the cell membranes and complex particles may explain this result. The positively charged complexes increased the electrostatic interactions between the cells and complexes. The results of confocal microscopy were consistent with the flow cytometry results.

### 3.5. Cytokine Induction by the ε-PLL/G4-CpG ODN Complexes at Various N/P Ratios

**Figure 6:**
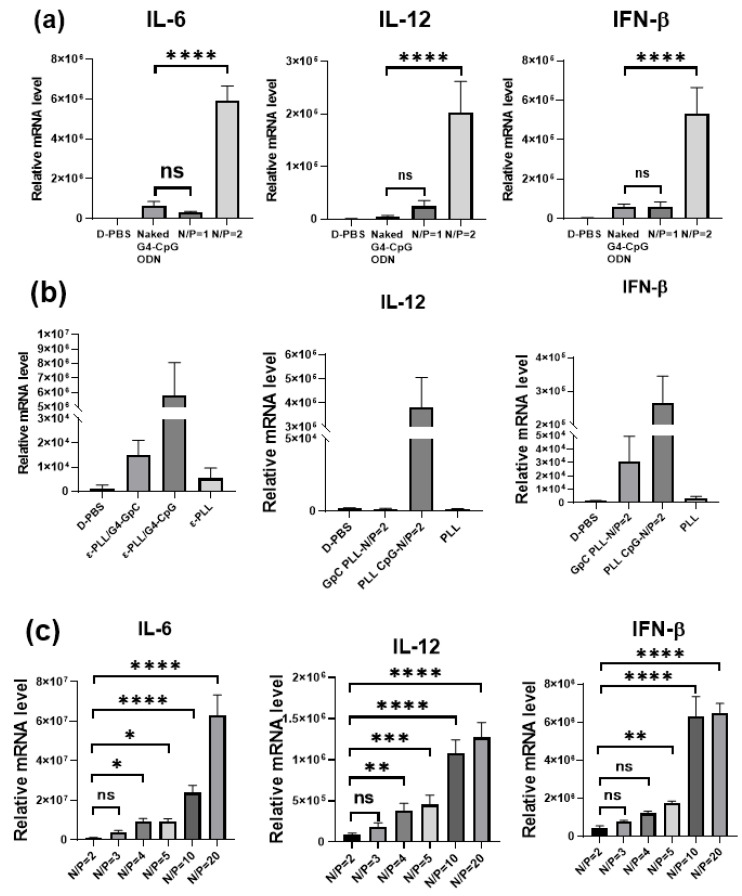
Cytokine induction in mouse macrophage-like RAW264 cells stimulated with the ε-PLL/G4-CpG ODN complexes. Relative mRNA levels in mouse macrophage RAW264 cells after 24 h of stimulation were measured. (**a**) ε-PLL/G4-CpG ODN complexes enhance cytokine production in RAW264 cells. (**b**) Effect of the CpG motif on cytokine production. N/P ratio of the complexes is 2. (**c**) Influence of the N/P ratios of the ε-PLL/G4-CpG ODN complex on cytokine production. The RAW264 cells were stimulated with the ε-PLL/G4-CpG ODN complexes at N/P ratios of 2, 3, 4, 5, or 20. The final G4-CpG ODN or G4-GpC ODN concentration is 1.25 μM. Dulbecco’s phosphate-buffered saline (D-PBS) is the negative control. Data represent mean ± SD (*n* = 5). **** *p* < 0.0001, *** *p* < 0.001, ** *p* < 0.01, * *p* < 0.5, ns: not significantly different (one-way ANOVA).

The CpG ODNs activate immune responses by secreting inflammatory cytokines such as tumor necrosis factor (TNF)-α, IL-6, IL-12, and type I IFNs via TLR9. In a previous study, we found that monomeric G4-CpG ODNs had increased immunostimulatory properties [24]. We investigated the immunostimulatory activity of ε-PLL/G4-CpG ODN complexes at N/P ratios of 1 and 2. As shown in Figure 6a, there was no significant difference between naked G4-CpG ODN and the complex with an N/P ratio of 1, which is attributed to the negative charge of the complexes at an N/P ratio of 1, similar to naked G4-CpG ODN. However, the ε-PLL/G4-CpG ODN complex at an N/P ratio of 2 induced significantly higher levels of IL-6, IL-12, and IFN-β production. The relative mRNA levels of IL-6 and IFN-β induced by the complex at N/P ratio 2 were five times higher than those induced by naked G4-CpG ODN, and the relative mRNA level of IL-12 was 10 times higher than that of G4-CpG ODN. The positive charge of ε-PLL/G4-CpG ODN at an N/P ratio of 2 provided high cellular uptake, further causing an increase in cytokine induction.

To estimate the immunostimulatory activity of ε-PLL, we evaluated the immunostimulatory activity of the complex with G4-GpC ODN, a sequence with no immunostimulatory capacity. Figure 6b shows that the ε-PLL/G4-GpC ODN complex did not induce cytokine secretion. These results indicate that ε-PLL itself has no immunostimulatory properties. The CpG motif of the G4-CpG ODN in the complex is recognized by TLR9, thereby promoting an immune response.

Furthermore, we investigated the effect of the N/P ratio on immunostimulatory activity. The relative mRNA levels of IL-6, IL-12, and IFN-β after the treatment of RAW264 cells with the complexes at various N/P ratios are shown in Figure 6c. We found that the relative mRNA levels of IL-6, IL-12, and IFN-β were positively correlated with the N/P ratios. Cells treated with the ε-PLL/G4-CpG ODN complex at an N/P ratio of 20 showed the highest levels of IL-6, IL-12, and IFN-β. The complex with an N/P ratio of 20 formed a particle size of 150 nm, which was smaller than those of the other complexes. A previous study showed that particle sizes ranging from 20 to 200 nm provide the most efficient cellular uptake rate [43]. The cellular uptake effect was responsible for the high immunostimulatory activity of the ε-PLL/G4-CpG ODN complex with an N/P ratio of 20. Moreover, the results indicate that cytokine induction levels could be easily tuned by changing the N/P ratio.

**Figure 7:**
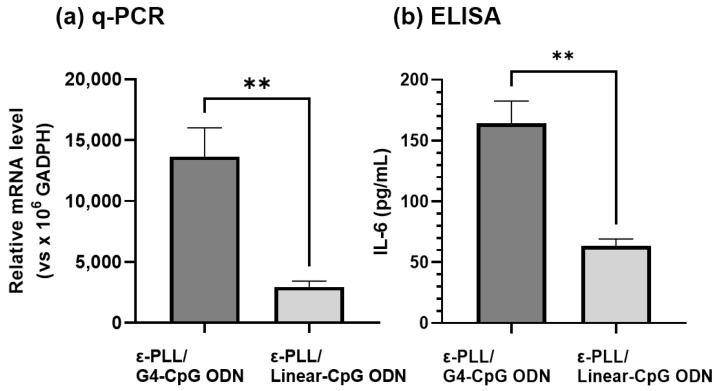
Immunostimulatory effect of the G4 structure in the complex on IL-6 induction. (**a**) Relative mRNA levels and (**b**) IL-6 protein expression level in mouse macrophage-like RAW264 cells after 24 h of stimulation. Data represent mean ± SD (*n* = 5). ** *p* < 0.01 (Student’s *t*-test).

We also compared the immunostimulatory effects of ε-PLL/G4-CpG ODN and ε-PLL/linear-CpG ODN. The ε-PLL/linear-CpG ODN complexes with an N/P ratio of 10 were prepared using the same methods. The final concentration of linear-CpG ODN in the medium was 1.25 μM, similar to that of G4-CpG ODN. As shown in Figure 7, ε-PLL/G4-CpG ODN induced greater amounts of IL-6 from RAW264 cells than ε-PLL/linear-CpG ODN. These results suggest that the G4 structure has a positive effect on the ε-PLL/ODN complex formation, resulting in enhanced immunostimulatory activity.

### 3.6. Adjuvant Effect of the ε-PLL/G4-CpG ODN Complexes

**Figure 8:**
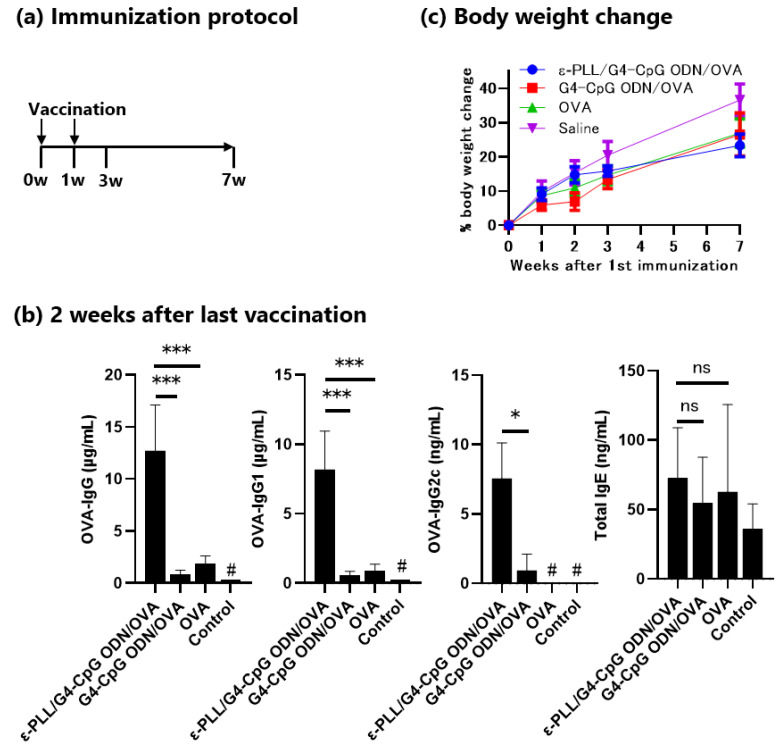
Adjuvant effects of the ε-PLL/G4-CpG ODN/OVA complexes in mice. (**a**) Immunization protocol. ε-PLL/CpG ODN was injected subcutaneously into mice (*n* = 4) twice at a 1-week interval. Seven weeks after the first vaccination, mice were boosted intraperitoneally with OVA. (**b**) OVA-specific IgG, IgG1, and IgG2c levels in serum 2 weeks after the second vaccination. (**c**) Effect of ε-PLL/G4-CpG ODN/OVA complexes on mouse weight gain. Data represent mean ± SD (*n* = 4). *** *p* < 0.001, * *p* < 0.5, ns: not significantly different (one-way ANOVA). ^#^ Denotes the detection limit (OVA-IgG and OVA-IgG1, 3.3 ng/mL; OVA-IgG2c, 0.39 ng/mL).

We evaluated the in vivo vaccine adjuvant effects of ε-PLL/G4-CpG ODN in mice. Mice were immunized with ε-PLL/G4-CpG ODN/ovalbumin (OVA) complexes, a mixture of G4-CpG ODN/OVA, or OVA alone via subcutaneous injection on the back twice at 1-week intervals. Two weeks after the second immunization, OVA-specific total IgG, IgG1, IgG2c, and total IgE antibodies in the serum were measured by ELISA. As shown in Figure 8, mice administered ε-PLL/G4-CpG ODN/OVA complexes showed significantly higher levels of OVA-specific total IgG, IgG1, and IgG2c antibodies than those administered a mixture of G4-CpG ODN/OVA and OVA alone. However, there was no significant difference in the level of total IgE between mice treated with the PLL/G4-CpG ODN/OVA complexes. The weights of the vaccinated mice were observed after the first vaccination. Mice injected with saline showed the highest weight gain, but there was no difference in weight gain among the three groups injected with the OVA antigen. These results indicate that ε-PLL/G4-CpG ODNs are potent vaccine adjuvants that induce antigen-specific antibodies without any side effects.

## 4. Discussion

As potential vaccine adjuvants, non-modified, entirely phosphodiester backbone-based CpG ODNs still have several deficiencies, such as easy degradation by nucleases and inefficient cellular uptake [19,20]. To overcome these limitations, various approaches have been developed to increase the enzymatic stability of CpG ODNs. In our previous study, we transformed linear-CpG ODNs into a structured CpG ODN named G4-CpG ODN, which showed high nuclease resistance; however, the hydrophilicity and negative charge of the G4-CpG ODN severely affected the efficiency of cellular uptake [23]. In the present study, we used the cationic polymer ε-PLL as a nanocarrier to condense G4-CpG ODNs and speculated that it could increase the cellular uptake of CpG ODNs through electrostatic interactions.

The ε-PLL/G4-CpG ODN complexes were formed by mixing ε-PLL and G4-CpG ODN solutions. The size of the ε-PLL/G4-CpG ODN complex can be controlled by the N/P ratio, and particles less than 200 nm in size suitable for cellular uptake can be prepared with a high N/P ratio. The zeta potential values were maintained at approximately +5 mV, and the N/P ratio increased to 20. This result is consistent with a previous study in which Bhatt et al. reported a charge of +4.47 mV for the ε-PLL aqueous solution [44].

Compared to naked G4-CpG ODNs, the presence of ε-PLL increased the nuclease resistance of G4-CpG ODNs. This result is consistent with that of a previous study showing that positively charged polyamines contribute to the stability of G4 structures [42]. Notably, when the N/P ratio reached 4, the percentage of residual CpG ODNs started to decrease. The lowest percentage of residual ODNs was obtained at an N/P ratio of 20. This may be because of the additional free cationic polymer. Gary et al. confirmed that cationic polymers and siRNA binding are reversible [42]. Above the critical charge-neutralization N/P ratio, the amount of cationic polymers increased with increasing N/P ratio. The abundance of free cationic polymers may lead to the condensation of CpG ODNs that are not tight. This may decrease the stability of the CpG ODNs.

ε-PLL increases the cellular uptake of G4-CpG ODNs. Because of the electrostatic repulsion between the negative charge of G4-CpG ODNs and the phosphate group of cell membranes, G4-CpG ODNs are difficult to uptake into cells. After condensation of G4-CpG ODNs by ε-PLL, enhanced cell uptake was observed because the cationic polymer neutralized the negative charge in G4-CpG ODNs. ε-PLL/G4-CpG ODN complexes easily bind to cell membranes and undergo endocytosis.

The high nuclease resistance and cellular uptake of ε-PLL/G4-CpG ODN complexes provide efficient immunostimulatory activity, which is easily observed by typical cytokine mRNA levels. When the N/P ratio was higher than 2, the relative mRNA levels increased with the increasing N/P ratio. The highest value was obtained at an N/P ratio of 20. However, an N/P ratio of 20 did not show the highest nuclease resistance. The suitable size of ε-PLL/G4-CpG ODN complexes formed at an N/P ratio of 20 and the reversible binding process may play an important role. The appropriate size of the complexes formed at an N/P ratio of 20 increased the possibility of internalization, and the flexibility of G4-CpG ODNs increased the number of G4-CpG ODNs available for TLR9 binding.

The ε-PLL/G4-CpG ODN complex showed an adjuvant effect in mice when administered with an antigen protein. In animal experiments, considering the particle size and cytokines induced from cells in vitro, ε-PLL/G4-CpG ODN complexes at an N/P ratio of 10 were used. Complexes with N/P ratios of 10 and 20 were less than 200 nm in size and induced large amounts of IL-6, IL-12, and IFN-β. Because the IL-12 and IFN-β levels were saturated at an N/P ratio of 10, we chose an N/P ratio of 10 for a preliminary in vivo experiment. Here, we showed that G4-CpG ODN, a non-modified ODN, induces antigen-specific IgG production. Non-modified nucleic acids do not function as adjuvants because they are easily degraded in the body. Many studies of CpG ODNs have used phosphorothioate-modified ODNs. Various types of carrier molecules, such as liposomes [45] and calcium phosphate [46], have been reported to show vaccine effects in combination with entirely or partially phosphorothioate-modified CpG ODNs; however, to the best of our knowledge, there are no reports on the use of non-modified, entirely phosphodiester backbone-based CpG ODNs. Furthermore, ε-PLL/G4-CpG ODN/OVA complexes induced both OVA-specific IgG1 and IgG2c, which correspond to Th2- and Th1-type responses associated with a subset of CD4^+^ T cells. The stronger antibody-dependent cellular cytotoxicity of IgG2 than that of IgG1 [47,48] suggests that the ε-PLL/G4-CpG ODN complex is highly likely to show vaccine effectiveness.

In summary, we developed a novel nanoscale complex of G4-CpG ODN and ε-PLL and showed that ε-PLL is an effective delivery carrier for CpG ODNs. ε-PLL exhibited both strong biocompatibility and a positive net charge, which improved the efficiency of the negatively charged G4-CpG ODNs. The size of the ε-PLL/G4-CpG ODN complex can be controlled by the N/P ratio, and particles less than 200 nm in size, suitable for cellular uptake, can be prepared with a high N/P ratio. Notably, the ε-PLL/G4-CpG ODN complexes exhibited strong cellular uptake and nuclease resistance. More importantly, ε-PLL/G4-CpG ODN complexes induced extremely high cytokine expression levels when using a small amount of CpG ODNs. Moreover, cytokine induction levels can be easily tuned by changing the N/P ratio. In addition, ε-PLL/G4-CpG ODN complexes showed excellent adjuvant effects in mice and potentiated antigen-specific antibody production. These results indicate that ε-PLL/G4-CpG ODN complexes have a great potential as vaccine adjuvants. We believe that our study improves the possibility of using G4-CpG ODNs as vaccine adjuvants in the clinical setting.

## Figures and Tables

**Table 1:** Formulation of the ε-PLL/G4-CpG ODN Complexes with Different N/P Ratios.

N/P Ratio	1	2	3	4	5	10	20
The concentration of ODNs in the complex solution	12.5 μM (149 μg/mL)						
The concentration of ε-PLL in the complex solution (μg/mL)	60	120	180	240	300	600	1200

## Data Availability

Not applicable.

## Supplementary Materials

The following supporting information can be downloaded at: https://www.mdpi.com/article/10.3390/biom12121868/s1. Figure S1: UV-vis spectra of ε-PLL. Figure S2: Polyacrylamide gel electrophoresis analysis of G4-CpG ODNs in the ε-PLL/G4-CpG ODN complexes. Figure S3: Intensity-, volume-, and number-weighted particle size distributions of the ε-PLL/G4-CpG ODN complexes at various N/P ratios. Figure S4: Scanning electron microscopy images of the ε-PLL/G4-CpG ODN complexes at an N/P ratio of 10. Figure S5: Serum stability of free G4-CpG ODN or ε-PLL/G4-CpG ODN complexes with various N/P ratios in 50% fetal bovine serum. Figure S6: Histogram analysis of fluorescence intensity at 4 °C and 37 °C. Table S1: Polydispersity index of the ε-PLL/G4-CpG ODN complexes by cumulants analysis.
