# Supplementary material for: Non-Modified CpG Oligodeoxynucleotide Forming Guanine-Quadruplex Structure Complexes with ε-Poly-L-Lysine Induce Antibody Production as Vaccine Adjuvants
Source: Biomolecules. 2022 Dec 13;12(12):1868. doi: 10.3390/biom12121868 (PMC9775190; doi:10.3390/biom12121868)
Supplement: Supplementary file 1 [file biomolecules-12-01868-s001.zip › biomolecules-1997424-supplementary.pdf]

Supporting Information

Non-modified CpG oligodeoxynucleotide forming guanine-quadruplex structure complexes with ε-poly-L-lysine induce antibody production as vaccine adjuvants

Dandan Zhao [1], Anh Thi Tram Tu [1,2,3,4], Miwako Shobo [1], Nguyen Bui Thao Le [1,2], Chiaki Yoshikawa [1], Kazuhisa Sugai [5], Yoji Hakamata [5] and Tomohiko YAMAZAKI [1,2,*]

[1.] Research Center for Functional Materials (RCFM), National Institute for Materials Science (NIMS), 1-2-1, Sengen, Tsukuba 305-0047, Japan

[2.] Division of Life Science, Hokkaido University, Kita 10, Nishi 8, Kita-ku, Sapporo 060-0808, Japan

[3.] Department of Magnetic and Biomedical Materials, Faculty of Materials Science and Technology, University of Science, Vietnam National University, 227 Nguyen Van Cu street, Ward 4, District 5, Ho Chi Minh City 70000, Vietnam

[4.] Vietnam National University, Linh Trung ward, Thu Duc city, Ho Chi Minh City 70000, Vietnam

[5.] School of Veterinary Nursing and Technology, Nippon Veterinary and Life Science University, 1-7-1 Kyonancho, Musashino-shi, Tokyo 180-8602, Japan

Corresponding author: T. Yamazaki

E-mail: yamazaki.tomohiko@nims.go.jp; Tel.: +81-29-859-2345; Fax: +81-29-859-2449

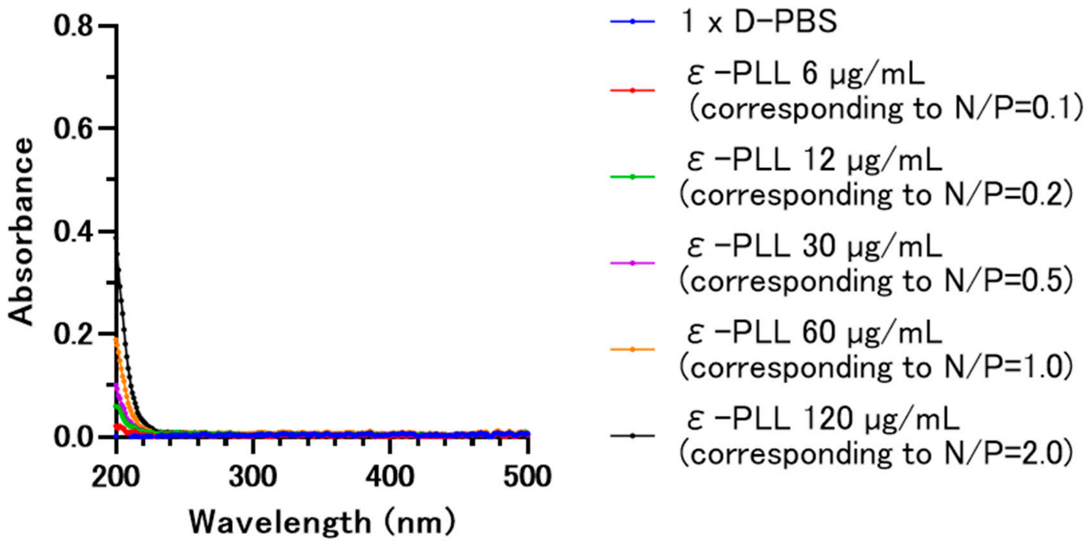

Figure S1. Ultraviolet-visible absorption (UV-vis) spectra of ε-PLL. UV-vis spectra of ε-PLL at concentrations of 6 μg/mL (red), 12 μg/mL (green), 30 μg/mL (purple), 60 μg/mL (orange), and 120 μg/mL (black). Each concentration corresponds to the final concentration of ε-PLL in the complexes at N/P ratios of 0.1, 0.2, 0.5, 1, and 2, respectively.

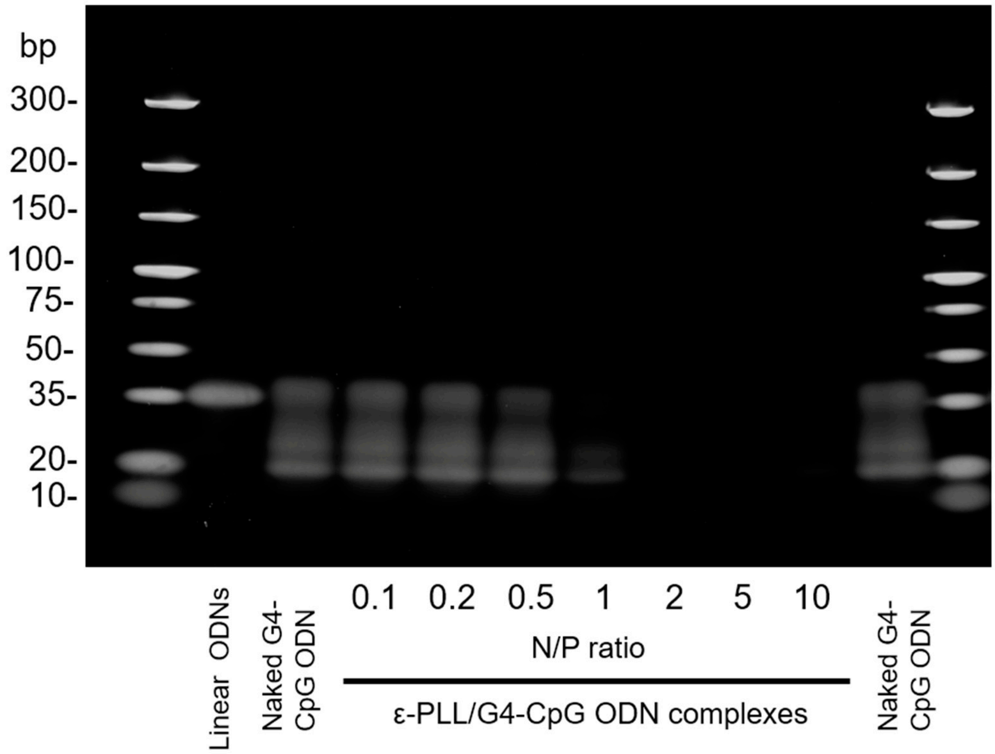

Figure S2. Polyacrylamide gel electrophoresis analysis of G4-CpG ODNs in the ε-PLL/G4-CpG ODN complexes. Electrophoresis was performed using a 10–20% linear gradient polyacrylamide gel in tris-glycine buffer supplemented with 4 mM KCl. Linear ODN is the ssODN38mer which has the same length as G4-CpG ODN (GD3).

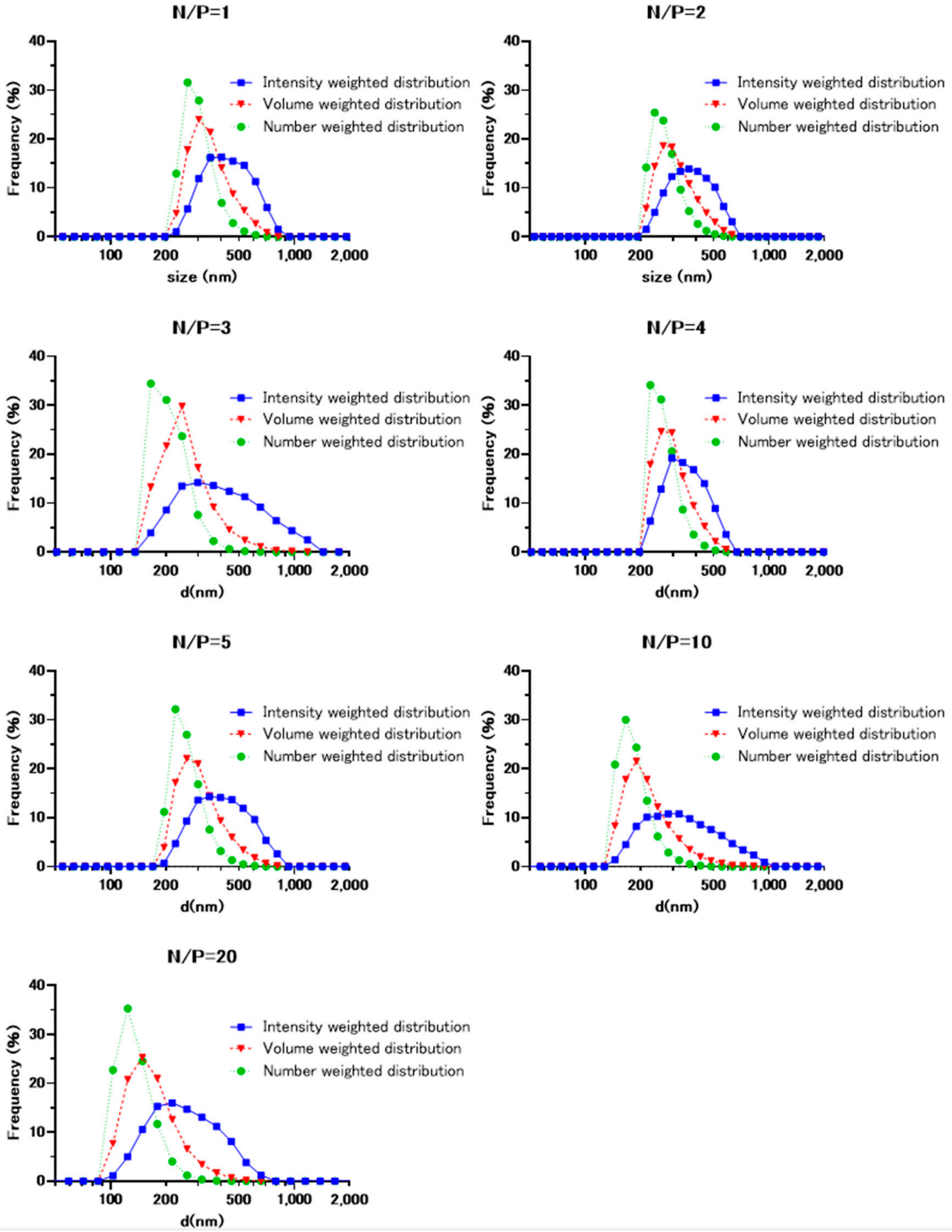

Figure S3. Intensity-, volume-, and number-weighted particle size distributions of ε-PLL/G4-CpG ODN complexes at various N/P ratios. Distribution was calculated using the Marquardt algorithm.

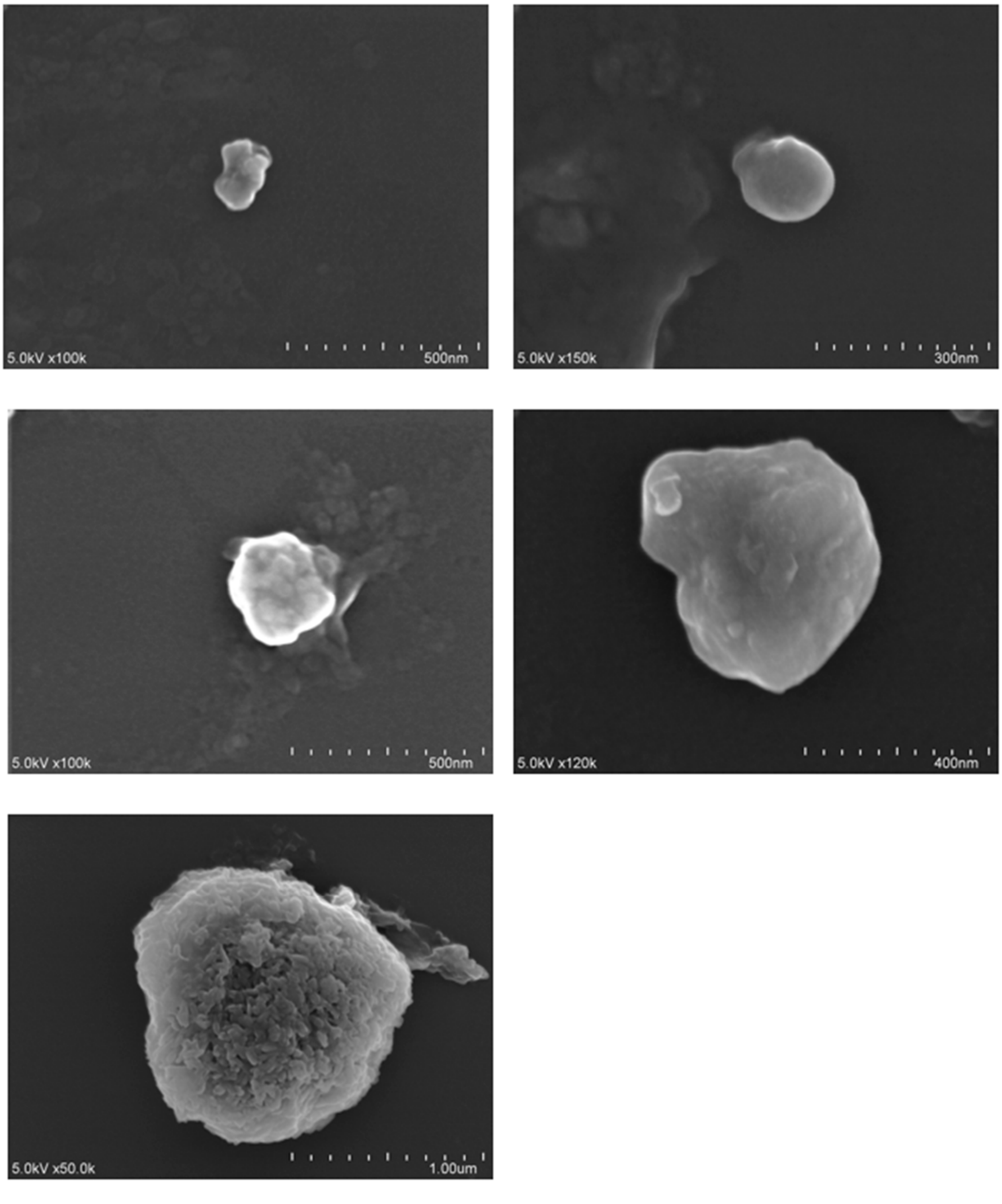

Figure S4. Scanning electron microscopy images of ε-PLL/G4-CpG ODN complexes at an N/P ratio of 10.

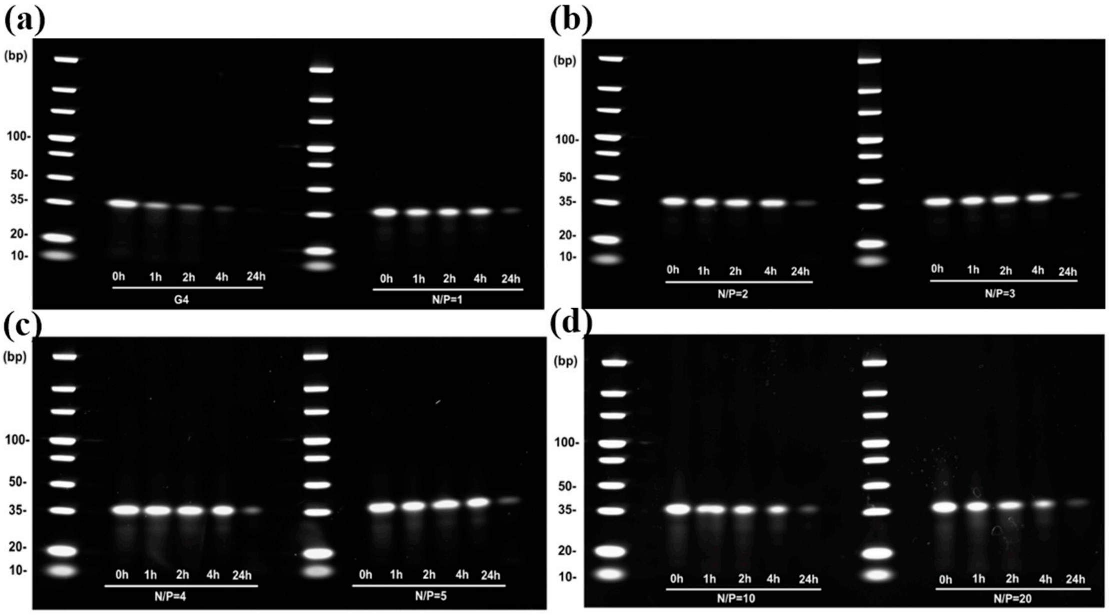

Figure S5. Serum stability of free G4-CpG ODN or ε-PLL/G4-CpG ODN complexes with various N/P ratios in 50% fetal bovine serum after incubation for 1, 2, 4, and 24 h. (**a**) naked G4-CpG ODNs and ε-PLL/G4-CpG ODN complex at N/P = 1, (**b**) ε-PLL/G4-CpG ODN complex at N/P = 2 and N/P = 3, (**c**) ε-PLL/G4-CpG ODN complex at N/P = 4 and N/P = 5, (**d**) ε-PLL/G4-CpG ODN complex at N/P = 10 and N/P = 20, as measured by polyacrylamide gel electrophoresis.

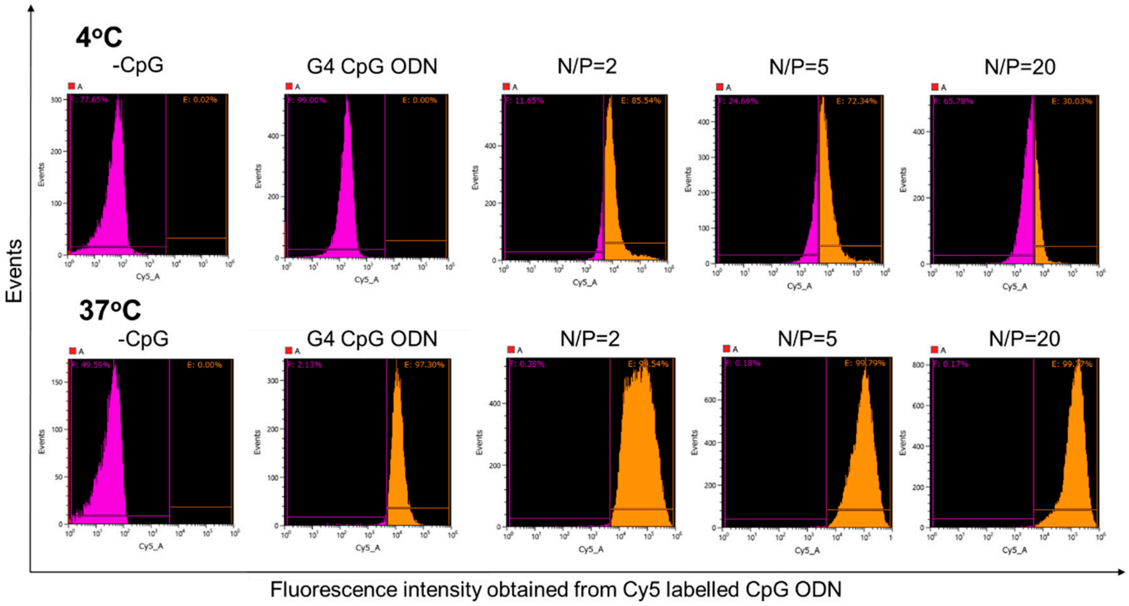

Figure S6. Histogram analysis of fluorescence intensity at 4 °C and 37 °C.

Table S1. Polydispersity index of ε-PLL/G4-CpG ODN complex by cumulants analysis

N/P ratio	1	2	3	4	5	10	20
PDI	0.141	0.026	0.182	0.111	0.145	0.094	0.156
